# Differential *In Vitro* Lung Cell Toxicity of Fresh and Photochemically Aged Smoke Aerosol Emissions from Simulated Wildland Fires of Duff and Surface Fuels

**DOI:** 10.1021/acsestair.5c00207

**Published:** 2025-09-17

**Authors:** Alexandra Noël, Chase K. Glenn, Omar El Hajj, Anita Anosike, Kruthika Kumar, Muhammad Isa Abdurrahman, Steven Flanagan, Mac A. Callaham, E. Louise Loudermilk, Elijah T. Roberts, Jonathan H. Choi, Bin Bai, Pengfei Liu, I. Jonathan Amster, Joseph O’Brien, Rawad Saleh

**Affiliations:** † School of Veterinary Medicine, Department of Comparative Biomedical Sciences, 5779 Louisiana State University, Baton Rouge, Louisiana 70803, United States; ‡ School of Environmental, Civil, Agricultural, and Mechanical Engineering, 1355 University of Georgia, Athens, Georgia 30602, United States; § USDA Forest Service Southern Research Station, Athens, Georgia 30602, United States; ∥ Department of Chemistry, University of Georgia, Athens, Georgia 30602, United States; ⊥ School of Earth and Atmospheric Sciences, 1372 Georgia Institute of Technology, Atlanta, Georgia 30332, United States

**Keywords:** wildfires, prescribed fires, atmospheric processing, particulate matter, oxidative stress, biotransformation

## Abstract

We investigated the effects of the fuel moisture content and photochemical aging on the toxicity of smoke particulate matter (PM) emissions in simulated wildland fires. We burned fuel beds consisting of surface fuels and duff under moderate and low moisture contents, representative of prescribed fires (Rx) and drought-induced wildfires (Wild), respectively. The Wild emissions were photochemically aged in an oxidation flow reactor (Wild-Aged). We exposed human bronchial epithelial cells to PM extracts from each permutation. PM extracts from all experimental permutations (Rx, Wild, Wild-Aged) induced oxidative stress, evidenced by a significant increase in 8-isoprostane concentration in the cell media compared to control. However, the increase of 8-isoprostane was significantly less in Wild-Aged compared to that in Wild and Rx, indicating loss of oxidative potential due to photochemical aging. Based on the release of lactate dehydrogenase in the cell media, the level of lipid peroxidation, and the magnitude of gene fold-changes, Rx PM extracts were more toxic than Wild. Chemical composition analysis suggests that toxicity was driven by levels of aromatic species in the PM, which were highest in Rx, followed by Wild and Wild-Aged. Overall, these results highlight the complex dependence of the toxicity of wildland-fire smoke on combustion conditions and atmospheric processing.

## Graphical abstract

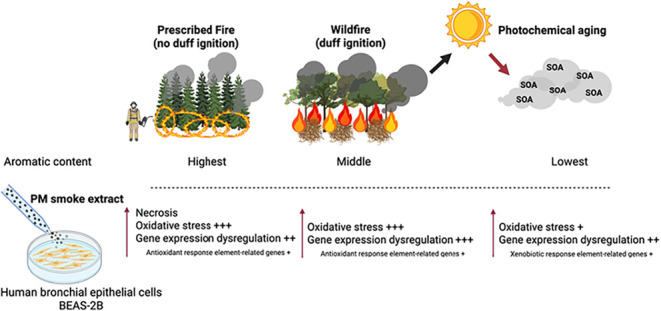

## 1 Introduction

Wildland fires are important for sustaining the ecological health of various forest types.^1,2^ Exposure to wildland-fire smoke (WFS), however, is an ever-growing public health threat resulting in ∼4000 premature deaths annually in the United States.^3^ Increasing epidemiological evidence demonstrates that respiratory and cardiovascular morbidities are the main health effects associated with exposure to WFS.^4^ Vulnerable populations such as children, the elderly, individuals with pre-existing respiratory diseases, and those from low socioeconomic status are at increased risk of pulmonary morbidity, including exacerbation, and mortality following exposures to WFS.^4^ Recent hospitalization data show a clear association between WFS exposures and asthma (significant relative risk or odds ratio ranging from 1.08 to 1.68).^5,6^ This is further illustrated by the 17% increase in emergency department visits for asthma observed in the U.S. during the April–August 2023 Canadian wildfire episode that reached the Northeast and Midwest continental U.S.^7^ These data clearly highlight that asthma is a pre-existing condition of concern during WFS episodes.

Wildland fires are categorized into wildfires and prescribed fires. Wildfires occur as unplanned ignitions due to lightning strikes and as the result of accidental human ignitions or arson.^8^ Prescribed fires are ignited intentionally for the purpose of forest management.^2^ Whereas wildfires can occur at a wide range of environmental conditions, they often take place during drought conditions when the fuel beds are dry.^9^ Prescribed fires, however, are usually ignited during favorable environmental conditions that fall within what is referred to as a prescription window, where the fuel beds are neither too dry nor too moist.^2,9^ The difference in environmental conditions between wildfires and prescribed fires leads to differences in fuel availability (the fraction of the fuel bed available for combustion),^9^ combustion conditions, and consequently, the quantities and chemical composition of the smoke emissions.^10,11^ This is especially the case for fuel beds that contain duff,^12^ a layer of partially decomposed forest litter that accumulates in regions that have undergone long periods of fire exclusion. By design, prescribed fires aim to consume surface fuels while excluding or minimizing the ignition of duff, and thus typically take place within a few days after rain in regions where the forest floor contains duff^12^ such that the duff is too moist to ignite. However, duff can ignite during droughts and can be consumed by drought-induced wildfires.^13,14^ A notable example is fall 2016, where a prolonged period of extreme drought led to extensive wildfires in the Southern Appalachian (Blue Ridge) Mountains^15^ that consumed large amounts of duff.^13^ Due to its compactness and lower carbon content compared to surface fuels, duff combusts less efficiently and exhibits more smoldering combustion compared to surface fuels.^11,16^ Even though smoldering combustion emits higher levels of particulate matter (PM), specifically organic aerosol (OA), compared to high-temperature (flaming) combustion, there is evidence that the smoldering PM is less toxic than flaming PM on a per-unit-mass basis.^17^ However, the dependence of PM toxicity on combustion conditions has not been investigated in the context of differences between drought-induced wildfires that feature duff ignition and prescribed fires that exclude duff ignition.

OA is the major PM constituent in WFS and biomass-burning smoke in general,^18–21^ and is the most complex as it features a myriad of species with highly variable chemical composition and physicochemical properties.^11,22–24^ Furthermore, wildland-fire smoke OA evolves significantly as it ages in the atmosphere due to formation of secondary organic aerosol (SOA) associated with gas-phase oxidation of organic species followed by gas-to-particle conversion, as well as—though to a lesser extent—heterogeneous oxidation of the primary OA (POA) emissions.^25–29^ Using the dithiothreitol (DTT) chemical assay, Jiang and Jang^30^ found that photochemical aging of woodsmoke for several hours in a smog chamber led to a significant decrease in the oxidative potential of the PM, which was attributed to dissociation reactions of the oxidizers in the PM. Similarly, Wong et al.^31^ performed laboratory experiments and found that oxidation with hydroxyl (OH) radicals in the aqueous phase led to a significant decline in the oxidative potential of biomass-burning PM. However, in their samples collected from the field, the same study found that the oxidative potential of wildland-fire PM increased by 50% after aging for several hours in the atmosphere and stabilized with further aging. We previously showed using an *in vitro* model that photochemical aging of smoke PM in a smog chamber led to more cell death by apoptosis, but less reduction in metabolic activity in lung epithelial cells compared to the fresh smoke PM.^32^ We attributed this differential response of lung epithelial cells to fresh and aged PM to differences in chemical composition, where the fresh PM featured higher levels of aromatic species and heavy metals, and the aged PM featured higher levels of oxygenated species. These data highlight the complexity of the effect of atmospheric photochemical aging on the toxicity profile of WFS and the need for a more detailed investigation of biological outcomes to develop a more complete understanding of how atmospheric aging impacts WFS toxicity.

The current study involved two goals. First, we explored how the differences in fuel moisture content in prescribed fires and drought-induced wildfires in regions where the forest floor contains duff affect the chemical composition of the emitted smoke PM and, consequently, its *in vitro* toxicity. To that end, we performed combustion experiments that simulated either a prescribed fire or a drought-induced wildfire using fuel beds that contained duff and surface fuels collected from a region in the Blue Ridge Mountains in the vicinity of the 2016 wildfires referenced above.^15^ Second, we explored whether and how the evolution in the chemical composition of smoke PM due to photochemical aging in the atmosphere led to changes in its *in vitro* toxicity. To that end, we simulated atmospheric photochemical aging in an oxidation flow reactor (OFR). We conducted comparative toxicity assessments of the various smoke PM extracts mentioned above in a human bronchial epithelial cell line (BEAS-2B cells). Biological outcomes included cytotoxicity, oxidative stress responses, and transcriptomic alterations.

## 2 Methods

### 2.1 Overview

The measurements and analyses presented in this paper were performed as part of the Georgia Wildland-fire Simulation Experiment (G-WISE), which took place in October-November 2022 at the U.S. Forest Service Southern Research Station Prescribed Fire Science Laboratory on the campus of the University of Georgia in Athens, GA. The combustion experiments in G-WISE were conducted using fuel beds constructed from fuels collected from three ecoregions in the Southeastern U.S.:^33^ Piedmont, Coastal Plain, and Blue Ridge Mountains. This study focuses on experiments conducted using Blue Ridge fuel beds. Whereas all fuel beds contained surface fuels, including fine and woody fuels, Blue Ridge was the only fuel bed that also contained a duff layer underneath the surface fuels. We conditioned the fuel beds to moisture contents representative of either prescribed fires (Rx) or drought-induced wildfires (Wild).^11,34^ Importantly, duff was not available for combustion under Rx conditions; thus, the smoke in the Rx experiments was produced from the combustion of surface fuels only. However, under Wild conditions, duff was available for combustion and dominated the smoke emissions.^11,34^ We note that prescribed fires purposefully exclude duff ignition^2,12^ but duff can be consumed in drought-induced wildfires.^13^ Therefore, our experiments replicated real-life differences between prescribed fires and drought-induced wildfires in regions in which the forest floor contains duff.

As further elaborated in Section 2.2, we collected PM samples for chemical composition and toxicity analyses from the fresh emissions from the Rx and Wild experiments. For the Wild experiments, we also collected samples from emissions after photochemical aging in an oxidation flow reactor (OFR). Consequently, this study involved three experimental permutations (Rx, Wild, and Wild-Aged), where the effect of combustion conditions was investigated by contrasting Rx and Wild, and the effect of atmospheric photochemical aging was investigated by contrasting Wild and Wild-Aged.

### 2.2 Combustion Experiments

The experiments are described in detail in previous G-WISE publications.^11,34^ Briefly, we used fuels collected from the Chattahoochee National Forest in the Blue Ridge Mountains to construct fuel beds that replicated the average mass loadings and three-dimensional (3D) structures observed in the field.^35–37^ The dry mass of the surface fuels was approximately 0.2 kg and consisted of 63% litter and 37% woody fuels. The dry mass of the duff was approximately 2.5 kg. For the Rx experiment, the moisture contents of litter, woody fuels, and duff were 10%, 36%, and 50%, respectively. The moisture content of the fine fuels was chosen to be within the values recommended by the U.S. Forest Service for prescribed fires in southern ecosystems (8%–15%).^38^ The higher moisture content of the woody fuels accounts for the time lag to adjust to atmospheric conditions, based on our field observations of prescribed fires. In regions that contain duff, prescribed fires are typically executed shortly after rainfall, such that the duff is too moist to ignite.^39^ The duff was collected 2 days after rainfall and had a moisture content of 50%. For the Wild experiment, the moisture content was less than 3% for all fuel components, which is representative of drought conditions that lead to duff ignition in wildfires.^13^

The combustion experiments were performed in a 990 m^3^ burn room. The fuel consumption was quantified in real time using a scale placed underneath the fuel beds, and the fire behavior was monitored in real time using thermal imagery.^11^ The mass consumption measurements and thermal imaging allowed for the determination of completion of the burns, after which smoke was sampled to an adjacent instrument room for various online measurements as well as filter collection for offline analyses. For the purposes of this study, the only online instrument utilized was the scanning mobility particle sizer (SMPS, TSI) to measure the particle size distributions. The filter collection procedure and sample preparation for chemical composition and toxicity analyses are described in Section 2.2.

The smoke emissions from the Wild experiment were oxidized with hydroxyl (OH) radicals in a potential aerosol mass oxidation flow reactor (PAM-OFR, Aerodyne Research)^40^ in order to simulate photochemical aging in the atmosphere. The details of the operation of the OFR during G-WISE are given in Deegan et al.^41^ The OH exposure estimated by OFR software based on the parametrization of Li et al.^42^ was 1.5 × 10^11^ molecules cm^–3^ s, which corresponds to 1.7 days of equivalent atmospheric aging, assuming an average OH concentration in the atmosphere of 10^–6^ molecules cm^–3^.^43^ We estimated the relative increase in OA mass concentration due to aging in the OFR, which is commonly referred to as OA enhancement,^25,28^ from the difference in integrated SMPS aerosol concentrations between the fresh aerosol and aged aerosol. OA enhancement is closely related to, but not necessarily exactly equivalent to, SOA formation, as there are other phenomena that can contribute to the change in OA concentration during photochemical aging. For example, OH reactions of semivolatile organic compounds (SVOCs) in the vapor phase can lead to particle-to-vapor partitioning of these SVOCs to restore phase equilibrium,^44^ thus reducing the OA concentration.

### 2.3 Sample Preparation for Chemical and Toxicity Analyses

We collected smoke PM samples on 47 mm PTFE filters (0.2 μm, Sterlitech Corporation, PTU024750). The Rx and Wild filter samples were collected directly from the burn room, and the Wild-Aged samples were collected downstream of the OFR (Section 2.2). Sample preparation followed the same procedure as in our previous studies.^32,45^ The extraction process involved placing the filters in a glass vial filled with 10 mL of methanol and sonicating for 10 min. The undissolved suspension was then removed from the solution using a glass syringe with a metal Luer lock tip through a 13 mm Teflon filter (0.2 μm, Sterlitech Corporation, PTU021350). A small portion (∼100 μL) of the solution was used for chemical speciation (Section 2.4), and the rest was used for toxicity analysis (Section 2.5). We note that smoke PM is largely comprised of organic compounds, which typically constitute more than 95% of the PM mass.^18–21^ Furthermore, previous studies have shown that more than 90% of the organics in the smoke PM are soluble in methanol.^46,47^ Therefore, the methanol extraction procedure recovers the majority of the smoke PM mass, which is dictated by organics but misses insoluble PM species, such as elemental carbon. We measured the mass concentration of organic carbon in the solution using the NIOSH-870 protocol^48^ in an organic-carbon elemental-carbon (OCEC) analyzer (Sunset Laboratory Inc., model 4L). The process involved pipetting 200 μL of the solution on a prebaked Quartz filter punch and evaporating the methanol under a stream of ultrapure nitrogen before analysis in the OCEC analyzer.

To prepare the smoke PM extracts for toxicity analyses, we evaporated the methanol from the solution using a stream of ultrapure nitrogen and then reconstituted the PM solutions in deionized water with 1.5% dimethyl sulfoxide (DMSO). The mass concentration of PM organic carbon in the solution was 250 μg/mL. As further elaborated in Section 2.5, the *in vitro* exposures involved adding 600 μL of the PM solutions to 2.4 mL of cell media, resulting in an exposure dose of 50 μg/mL with 0.3% DMSO. Blanks were prepared by extracting clean filters using the same procedure as the PM samples.

### 2.4 Chemical Analysis

The methanol extracts of the OA fraction of the PM collected from Rx, Wild, and Wild-Aged were analyzed using electrospray ionization Fourier transform ion cyclotron resonance mass spectrometry (ESI-FTICR-MS) using the same procedure described in Glenn et al.^11^ ESI is widely used for analyzing biomass-burning OA^49–51^ because it is composed of molecules that are efficiently ionized by ESI.^52,53^ We performed the analysis in negative ionization mode using a Bruker SolariX XR 12 T FTICR mass spectrometer with an *m*/*z* range of 70–1000. The transient length was 1.667 s, yielding a mass resolution of ∼430,000 at *m*/*z* 400. The capillary was set to 4500 V with an end plate offset of −800 V. We maintained the dry gas rate at 4.0 L/min, the nebulizer gas pressure at 0.8 bar, and the dry temperature at 200 °C. We acquired three spectra for each sample, and each spectrum was an average of 48 scans.

We prepared a blank solution by extracting a clean filter using the same procedure described in Section 2.3. We obtained molecular assignments from the blank-subtracted mass spectra using MFassignR.^54^ The procedure involved initial carbon, hydrogen, and oxygen (CHO) assignments using a mass tolerance of 1 ppm, followed by identification and filtering of ^13^C and ^34^S isotopes, such that only monoisotopic peaks were selected. The monoisotopic peaks then underwent an internal mass recalibration,^55^ and the final assignments for recalibrated peaks were obtained based on a constraint that the number of nitrogen atoms is less than or equal to three. Sulfur-containing compounds constituted less than 2% of the assignments and were not considered in the analysis.

### 2.5 Toxicity Analyses

#### 2.5.1 Cell Culture and Exposure Conditions to Smoke PM Extracts

We used a human bronchial epithelial cell line (BEAS-2B cells, ATCC CRL-9609) to evaluate the *in vitro* toxicity of various smoke PM extracts generated in this study. We used T-75 tissue culture flasks to grow and culture the cells until ready for use. We cultured and maintained the BEAS-2B cells in DMEM medium with 10% fetal calf serum, 100 U/mL penicillin, and 10 μg/mL streptomycin, as previously described in Pinkston et al.^56^ The cells were placed in an incubator and were maintained at 37 °C in a 100% humidified atmosphere containing 5% CO_2_. For this study, we used cells from passages 12 to 16. Before the exposure, BEAS-2B cells were seeded onto 6-well tissue culture plates at a density of ∼5 × 10^5^ cells/well. The medium was changed every 2 days. 72 h post seeding, the cells were exposed to the smoke PM extracts by adding 600 μL of the PM solution (stock solution 250 μg/mL) in 2.4 mL of cell media. This resulted in an exposure dose of 50 μg/mL in each well. *In vitro* exposure doses recreating firefighters’ WFS exposure level were reported to be approximately 19 μg/cm^2^.^57^ Based on the surface area of a 6-well plate, the *in vitro* exposure dose used in our study was ∼15.6 μg/cm^2^, which highlights the relevance of our exposure dose in the context of WFS. The control cells were exposed to extracted blank filters, resulting in 0.3% DMSO. The cells were exposed to the smoke PM extracts for 24 h, and then the cells and the media were collected for analysis. Each exposure condition was evaluated using 5 biological replicates, each having 2 technical replicates.

#### 2.5.2 Cell Viability

We evaluated the cell viability *via* Trypan blue exclusion assay 24 h post exposure. Following cell collection, the cells were stained with trypan blue solution and placed on a TC10 counting slide (Catalog No. 1450015, Bio-Rad Laboratories, Hercules, CA). We then used a TC20 automated cell counter (Catalog #1450102, Bio-Rad Laboratories, Hercules, CA) to determine the cell viability. Each sample was read in duplicate.

#### 2.5.3 Caspase 3-Positive Cells

We used a Cellometer Spectrum System (SPECTRUM-SYS1–10) that includes two fluorescence optics modules from Nexcelom Bioscience LLC (Lawrence, MA) to evaluate the number of Caspase 3-positive cells from the BEAS-2B cells collected 24 h post exposure. We used the Caspase 3 assay from the Cell Fitness Panel for Cellometer Spectrum (catalogue no. CSK-V0023–1, Revvity) and followed the manufacturer’s instructions.

#### 2.5.4 Lactate Dehydrogenase (LDH) Assay

We measured LDH levels in the cell media collected 24 h post exposure. We followed the manufacturer’s instructions to conduct the assay (CyQuant, LDH cytotoxicity assay kit, Catalog No. C20300, Invitrogen, Thermo Fisher Scientific, Waltham, MA). We used a spectrophotometer (TECAN Infinite 2000) at a wavelength of 490 nm, with 630 nm set as the reference, to quantify LDH in cell media. For all samples, we normalized the absorbance to the total cell count. The data is expressed as a percentage from controls since the absorbance values of the control group were set at 100%. Each sample was read in duplicate.

#### 2.5.5 8-Isoprostane ELISA

We measured 8-isoprostane concentration in the cell media collected 24 h post exposure. As recommended by the manufacturer, we added butylated hydroxytoluene (BHT) at the time of the media collection. We followed the manufacturer’s instructions to conduct the 8-isoprostane ELISA (8-Isoprostane Express ELISA Kit, Catalog No. 516360; Cayman Chemical; Ann Arbor, MI). We used a spectrophotometer (TECAN Infinite 2000) to evaluate the absorbance at wavelengths of 405 nm–420 nm. The standard curve was established using the absorbance values for the standards, and the concentrations in the sample media were calculated. Each sample was read in duplicate.

#### 2.5.6 Griess Assay

We measured levels of nitric oxide (NO) in the cell media collected 24 h post exposure. We followed the manufacturer’s instructions and used the Griess reagent kit (Catalog #30100, Biotium, Fremont, CA). We used a spectrophotometer (TECAN Infinite 2000) to evaluate the optical density at a wavelength of 548 nm. For all samples, we normalized the optical density values to the total cell count. The data was expressed as a percentage from controls since the optical density values of the control group were set at 100%. Each sample was read in duplicate.

#### 2.5.7 RNA Extraction and qRT-PCR

We conducted RNA extraction following the cell collection 24 h post exposure. This was completed using the RNeasy Plus Mini Kit (Catalog No. 74136, Qiagen, USA). We used a NanoDrop 1000 (Thermo Scientific) (260/280 nm ratio) to evaluate the quantity as well as the quality of the RNA extracted. We prepared cDNA using the RNA-to-cDNA kit (Catalog No. 4387406, Applied Biosystems, USA). We used commercially available probe sets from Taqman (Applied Biosystems, University Park, IL). As described in Pinkston et al.,^56^ a selection of genes was run in an Applied Biosystems 7300 Real-Time PCR System. Cycle numbers were used to assess relative gene expression to controls based on the comparative cycle threshold (ΔΔCT) method. β-actin was used as the housekeeping gene. We expressed the results as fold-change over the control. Each gene evaluated from each sample was read in duplicate.

#### 2.5.8 Statistical Procedures

We used GraphPad Prism Software (GraphPad Software, San Diego, CA) to evaluate the statistical significance of all of the biological outcomes evaluated in the *in vitro* experiments. We used either the Student *t* test for pairwise comparisons or one-way ANOVA for the comparison of 3 or more groups, to determine the significance of the differences observed between the experimental groups and the controls. Results are expressed as mean ± standard error of the mean (SEM). Results were considered statistically significant with a *p* < 0.05 or for gene expression data, when a fold-change > |1.5|.

## 3 Results

### 3.1 Chemical Composition of Organic Aerosol Emissions

The OA molecules in the smoke PM extracts detected by ESI-FTICR-MS are mapped based on their O/C and H/C in the van Krevelen plots depicted in Figure 1. Figure 1a contrasts Rx and Wild by isolating the OA molecules that are unique to each and those that are common between the two. The OA molecules unique to Wild were relatively less oxidized (*i.e*., have lower O/C) compared to those unique to Rx. We attribute this result to duff being available for combustion in Wild but not in Rx, leading to different combustion conditions. Specifically, the compactness and relatively lower carbon content of duff compared to surface fuels^11,16^ led to less efficient (lower temperature) combustion in Wild compared to Rx. As illustrated in Figure 2, both Rx and Wild exhibit a high-temperature peak associated with the flaming phase of combustion, followed by a low-temperature tail associated with the smoldering phase. However, due to duff combustion, the smoldering phase in Wild was significantly longer than Rx. This low-temperature oxygen-deprived smoldering combustion is conducive to the formation of less oxidized molecules in Wild compared to Rx (Figure 1a). We note that the difference in combustion temperature between Wild and Rx (Figure 2) is representative of differences in real field combustion conditions between prescribed fires and drought-induced wildfires of fuel beds that contain duff.^10^ To further explore the effect of combustion conditions on OA formation, we calculated the fraction of OA molecular assignments that are classified as aromatic and condensed aromatic.^58^ As shown in Table 1, the OA molecular assignments in Rx had higher levels of aromatics and condensed aromatics compared to Wild. The formation of aromatic species is favored by high combustion temperatures,^59^ and is therefore consistent with the observation that Rx featured relatively higher temperatures compared to Wild (Figure 2).

**Figure 1:**
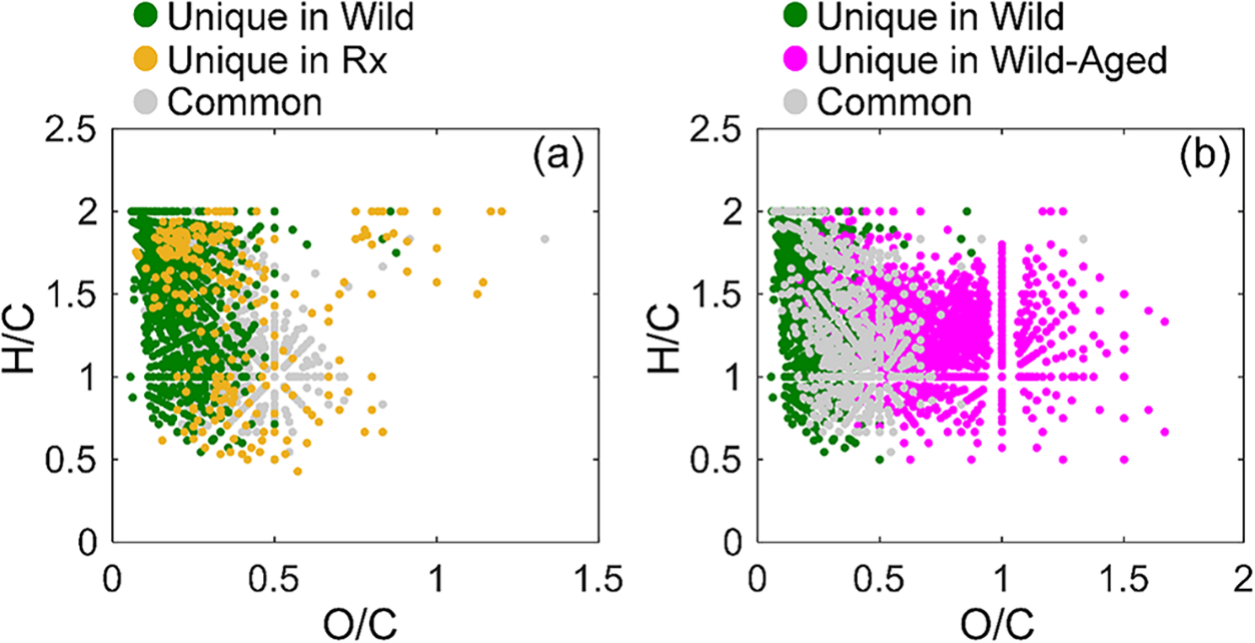
van Krevelen plots of the molecular assignments of OA species detected by using ESI-FTICR-MS. (a) Contrasts Rx and Wild. (b) Contrasts Wild and Wild-Aged. The gray dots correspond to molecules common between the two experimental permutations (Wild vs Rx and Wild vs Wild-Aged), and the other color dots correspond to molecules unique in each permutation.

**Figure 2:**
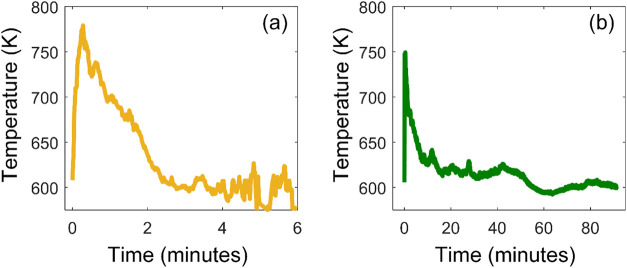
Average fuel bed fire temperature over the course of the burn for (a) Rx and (b) Wild. Note the difference in *x*-axis scales between panels.

**Table 1:** Fraction of Molecular Assignments of OA Species Detected by ESI-FTICR-MS That Are Classified as Aromatics and Condensed Aromatics for Each of the Experimental Permutations

comparison	permutation	fraction of aromatics	fraction of condensed aromatics
N/A	Rx	14.1%	3.7%
N/A	Wild	12.5%	2.3%
N/A	Wild-Aged	8.5%	0.9%
Wild vs Rx	Unique in Wild	9.8%	1.6%
	Unique in Rx	13.8%	7.5%
Wild vs Wild-Aged	Unique in Wild	10.3%	2.7%
	Unique in Wild-Aged	4.4%	0.5%

The effect of photochemical aging on the chemical composition of the OA (Wild vs Wild-Aged) is more prominent than the effect of differences in combustion conditions (Wild vs Rx). As shown in Figure 1b, the OA that was oxidized with OH radicals in the OFR (Wild-Aged) consisted of molecules that had substantially higher O/C compared to the fresh OA molecules (Wild). Furthermore, OA unique to Wild-Aged had a substantially lower fraction of molecular assignments classified as aromatic and condensed aromatic compared to Wild (Table 1). This finding indicates that the gas-phase SOA precursors included fewer aromatic species compared to POA.

Based on the SMPS measurements, the OA enhancement ratio (ratio of aged aerosol mass to fresh aerosol mass) in the OFR was approximately 1.75, which is on the high end compared to previous studies on photochemical aging of biomass-burning OA.^29^ We attribute this finding to the prominent smoldering associated with duff combustion. This inefficient low-temperature combustion is more conducive for the formation of relatively small organic molecules, which exist predominantly in the gas phase and are SOA precursors, compared to higher-temperature combustion that is more commonly encountered in biomass-burning studies.^27,28^ Consequently, smoke emissions from duff combustion have a relatively high SOA formation potential compared to those from other fuels. Therefore, we expect that the observed increase in molecular assignments with high O/C (Figure 1) and the decrease in fraction of aromatic and condensed aromatic species (Table 1) associated with aging in the OFR was largely driven by the high level of SOA formation. However, the changes in chemical composition, especially the increase in O/C, can also be in part due to heterogeneous chemistry.^29^

### 3.2 Rx Smoke PM Extracts Are Toxic to BEAS-2B Cells

BEAS-2B cells were exposed for 24 h to DMSO (control), Rx smoke PM extracts, Wild smoke PM extracts, or Wild-Aged smoke PM extracts, at a concentration of 50 μg/mL, which led to >75% cell viability in all groups (Figure 3). Although the decrease in cell viability between the DMSO control (82.6%) and the Rx smoke PM extracts (75.6%) was approaching significance (*p* = 0.0529) (Figure 3), the release of LDH from the BEAS-2B cells into the media was significantly increased following the 24 h exposure to the Rx smoke PM extracts (58% increase) compared to the DMSO control (Figure 4a). This highlights that Rx smoke PM extracts increased cytotoxicity mainly driven by necrotic cell death, as opposed to apoptosis. Indeed, LDH is released from cells following cell death due to the increased permeabilization of the plasma membrane.^60^ Thus, LDH is used as a marker for cell death by necrosis. In addition, using a Caspase 3 assay, we were able to exclude that apoptosis was the main mechanism of cell death since no significant difference for Caspase 3-positive cells was noted between the groups (Figure 4b). Caspase 3 is necessary for the efficient execution of apoptosis and can be used as an indicator of late-stage apoptosis.^61^ Overall, Rx smoke PM extracts were more toxic to BEAS-2B cells than the Wild- or Wild-Aged smoke PM extracts.

**Figure 3:**
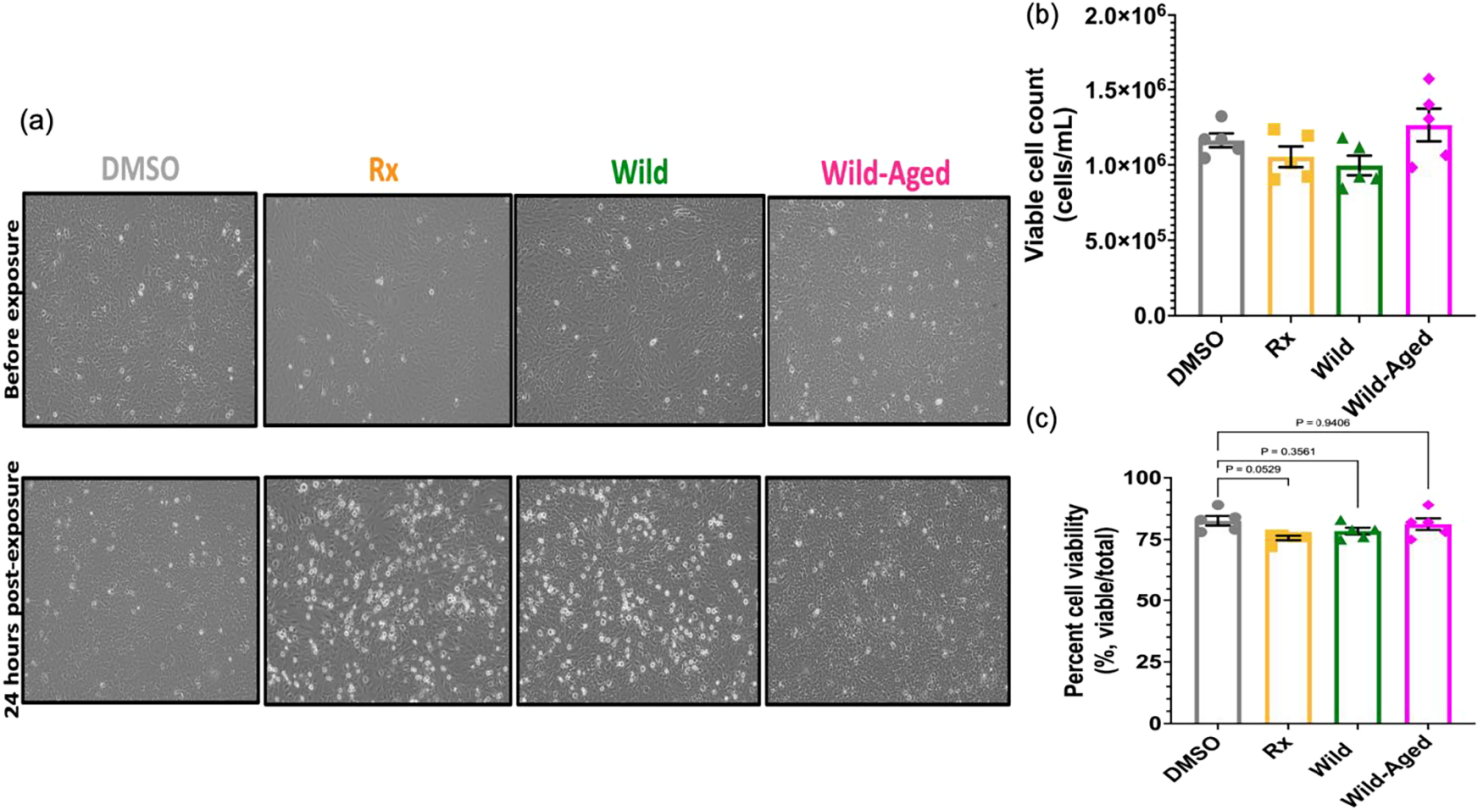
BEAS-2B cells were exposed for 24 h to smoke PM extracts. (a) Representative microscopy images of the BEAS-2B cells in the 6-well plate before exposure and 24 h post exposure (before cell and media collection). (b) Viable cell count 24 h post exposure. (c) Percentage of viable cells (viable cell count/total cell count) 24 h post exposure. Data are expressed as the mean ± SEM, *N* = 5 biological replicates per condition, each having *n* = 2 technical replicates.

**Figure 4:**
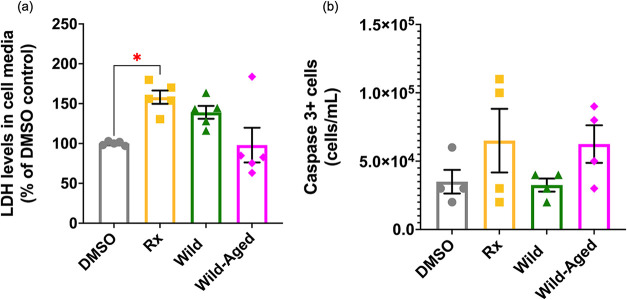
Exposure to Rx smoke PM extracts significantly increased the release of LDH in the cell media of BEAS-2B cells. (a) Levels of LDH measured in the cell media of the BEAS-2B cells 24 h post -smoke PM extract exposure. (b) Count of Caspase 3+ cells 24 h post smoke PM extracts exposure. Data are expressed as the mean ± SEM. Statistical significance: * *p* < 0.05. *N* = 4–5 biological replicates per condition, each having *n* = 2 technical replicates.

### 3.3 Rx, Wild, and Wild-Aged Smoke PM Extracts Induce Oxidative Stress Responses in BEAS-2B Cells

While Rx, Wild, and Wild-Aged smoke PM extracts significantly increased the concentration of 8-isoprostane (a biomarker of oxidative stress more specifically of lipid peroxidation) in the cell media (18.7–78.3 pg/mL) compared to the DMSO control (7.2 pg/mL) (Figure 5a), levels of 8-isoprostane were significantly higher following the exposure to Wild smoke PM extracts (73.8 pg/mL) compared to Wild-Aged smoke PM extracts (18.7 pg/mL) (Figure 5a). Albeit nonsignificant, we observed an increased percentage of NO levels in the cell media following Rx and Wild smoke PM extract exposures (Figure 5b), thus following a similar pattern as for oxidative stress responses. Although our results do not discriminate between neuronal NO (nNOS), endothelial NO (eNOS), or inducible NO (iNOS), NO in the bronchial epithelium, mainly iNOS, is involved in signaling pathways related to inflammation; thus, alteration of NO levels is detrimental to the bronchial epithelium barrier.^62^ Further, supporting our oxidative stress effect in the cell media, we observed at the molecular level a greater upregulation of antioxidant response element (ARE) genes following exposure to the Wild compared to the Wild-Aged smoke PM extracts (NQO1:5.1-fold vs 4.8-fold, and HMOX1:3.9-fold vs 3.6-fold, for Wild and Wild-Aged, respectively) (Figure 6). Moreover, ARE genes are regulated by the *Keap1/Nrf2* pathway, and KEAP1 was upregulated 1.5-fold only following the exposure to the Wild smoke PM extracts (Figure 6). In contrast, xenobiotic response element (XRE) genes, which are regulated by aryl hydrocarbon receptor (AHR) signaling, were upregulated to a greater extent by Wild-Aged smoke PM extracts (CYP1A1:16.4-fold vs 19.2-fold, CYP1B1:3.4-fold vs 4.1-fold, and ALDH3A1:3.3-fold and 6.0-fold, for Wild and Wild-Aged, respectively) (Figure 6). In total, 13 genes were significantly dysregulated following the exposure to Wild smoke PM extracts, whereas 10 genes were significantly dysregulated following the exposure to Wild-Aged smoke PM extracts (Figure 6). Comparing the dysregulated genes by exposure to Rx and Wild smoke PM extracts, there were 10 and 13 significantly altered genes, respectively (Figure 6). Exposure to Rx smoke PM extracts induced the greatest upregulated fold-change for CYP1A1 (24.8-fold). Also, XRE genes were slightly more upregulated following exposure to Rx versus Wild smoke PM extracts (CYP1B1:4.6-fold vs 3.4-fold, and ALDH3A1:3.7-fold vs 3.3-fold, for Rx and Wild, respectively) (Figure 6). Similar molecular results were observed for ARE dysregulated genes due to exposure to Rx and Wild smoke PM extracts (NQO1:5.2-fold vs 5.1-fold, and HMOX1:4.6-fold vs 3.9-fold, for Rx and Wild, respectively) (Figure 6). In addition, the exposure to smoke PM extracts from all three conditions downregulated the expression of inflammation-related genes, including IL-6 (−2.8 to −3.2-fold; Figure 6). These results suggest that in this model and at the concentration of 50 μg/mL, the smoke PM extracts seem to inhibit/suppress a pro-inflammatory response.

**Figure 5:**
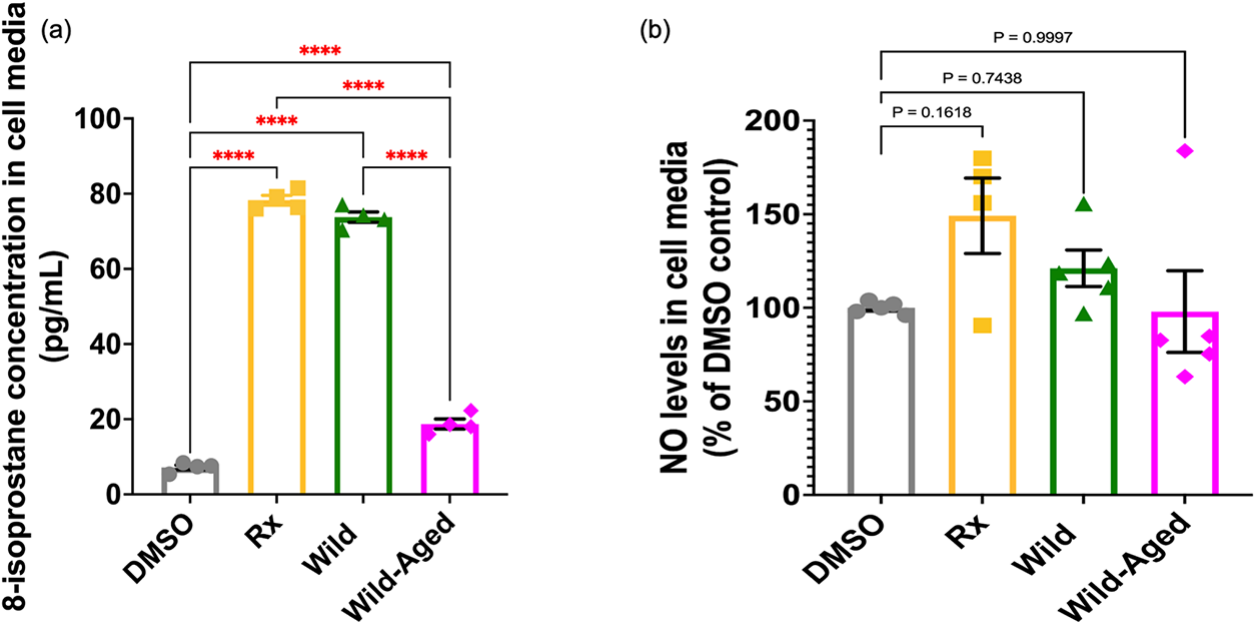
Exposure to Rx, Wild, and Wild-Aged smoke PM extracts significantly increased the release of 8-isoprostane in the cell media of BEAS-2B cells. (a) Concentrations of 8-isoprostane measured in the cell media of the BEAS-2B cells 24 h post exposure. (b) Levels of NO measured in the cell media of the BEAS-2B cells 24 h post exposure. Data are expressed as the mean ± SEM. Statistical significance: **** *p* < 0.0001. *N* = 4–5 biological replicates per condition, each having *n* = 2 technical replicates.

**Figure 6:**
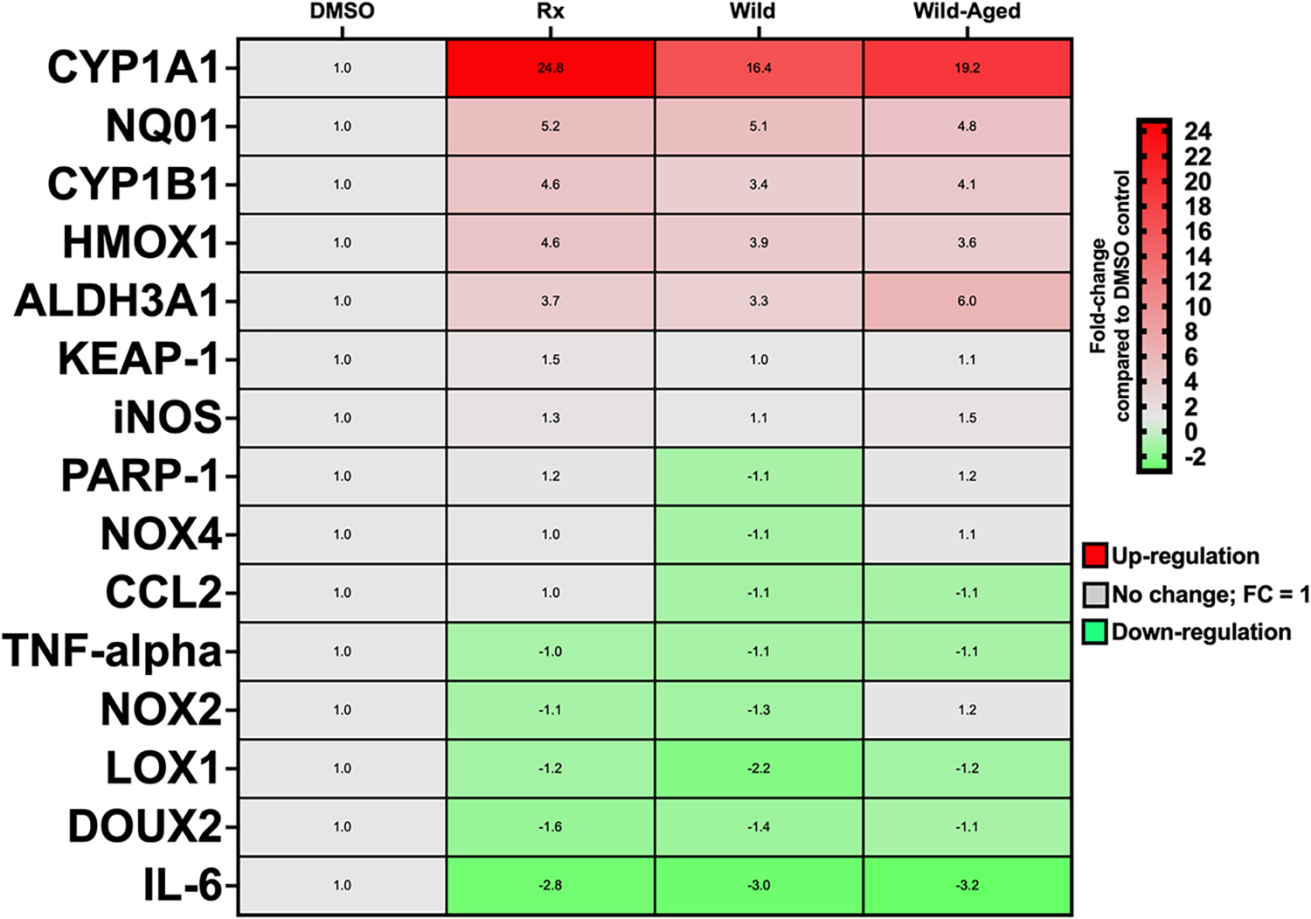
Exposure to Rx, Wild, and Wild-Aged smoke PM extracts dysregulated the expression of several genes associated with biotransformation, oxidative stress, and inflammation in BEAS-2B cells. The heatmap shows dysregulated genes by exposure to smoke PM extracts in BEAS-2B cells compared to DMSO controls. Data are expressed as fold-changed (noted in each cell) compared with DMSO controls. Significance was considered when fold-change > |1.5|.

Overall, the main molecular pathways affected by exposure to smoke PM extracts involve biotransformation and oxidative stress. Also, Rx smoke PM extracts dysregulated XRE and ARE-associated genes to a greater extent than Wild smoke PM extracts. In addition, our data suggest that AHR and *Keap1/Nrf2* signaling pathways, which are key transcription factors involved in oxidative stress responses and pulmonary pathogenesis,^63^ are differently modulated by Wild compared to Wild-Aged smoke PM extracts.

## 4 Discussion

### 4.1 Oxidative Stress as a Central Mechanism of Toxicity Induced by Exposure to Wildland-Fire Smoke

The central mechanisms by which WFS induce adverse health effects are associated with pathways related to cytotoxicity, inflammation, oxidative stress, redox imbalance, and genotoxicity.^64–66^ Further, dysregulated gene expression can lead to alterations in key signaling pathways within the cell, which can impact cell function^67^ and lead to two main cell death mechanisms, apoptosis and necrosis.^65,68^ In both cases, high levels of reactive oxygen species (ROS) within the cell can result in the oxidation of lipids or proteins, as well as DNA damage.^66^ A critical difference between those two cell death mechanisms relies on the integrity of the plasma membrane.^68^ In contrast to apoptosis, the programmed cell death mechanism, necrosis is evidenced by increased permeation of the plasma membrane, which leads to the release of LDH from the cytosol.^60^ Cell death, whether *via* apoptosis or necrosis, can be induced by a high presence of ROS, resulting in elevated oxidative stress levels within the cell.^69^ In our study, the Rx condition significantly increased the percentage of LDH released in the cell media compared to that of the DMSO controls, concurrent with no significant change seen for Caspase 3, a marker of late phase apoptosis (Figure 4). Thus, for the Rx condition, we observed necrosis after a 24 h exposure; however, a different response, for instance, late-stage apoptosis, could have been observed at an earlier time point, *e.g*., 6 h post exposure, which we did not evaluate. One study exposed human alveolar epithelial type II cells to woodsmoke and observed a significant increase in media LDH,^70^ similar to our findings for the Rx condition (Figure 4a). It was demonstrated that adverse biological outcomes and alterations of gene expression and cellular signaling pathways can arise without any significant effects on cell viability.^71^ This is consistent with our results since we found that the smoke PM extracts for the Rx condition increased LDH and 8-isoprostane levels (Figures 4 and 5), while slightly affecting the BEAS-2B cell viability (Figure 3). This indicates that significant alterations to the cells’ function, including potentially increased permeability, can be caused by woodsmoke-infused solution or smoke PM extract exposures in the absence of substantial cytotoxicity, suggesting that cytotoxicity may not necessarily be a sensitive indicator of woodsmoke or smoke PM extract toxicity in these *in vitro* contexts.

Our results highlight that smoke PM extracts from all experiments induced oxidative stress responses, as evidenced by the concentration of 8-isoprostane in the cell media (Figure 5) and the upregulation of oxidative stress-related genes, including NQO1 and HMO1 (Figure 6). These results are consistent with other similar studies, which also showed that wood tar extracts and woodsmoke particles caused lipid peroxidation in lung epithelial cells and in macrophages.^66,72^ In addition, activation of the aryl hydrocarbon receptor (AHR) signaling pathway by PM can significantly increase ROS production, and elevated oxidative stress levels can activate the antioxidant response element (ARE) canonical target genes, regulated by Keap1/Nrf2 signaling.^73,74^ Thus, the upregulation of genes part of the ARE signaling pathway is an indication of activated oxidative stress responses,^64^ and in our study, smoke PM extracts from all experiments upregulated the expression of genes associated with the ARE signaling pathway (Figure 6). Overall, our findings support the crucial role of oxidative stress in early cellular responses to smoke PM extracts, irrespective of exposure conditions.

### 4.2 Effect of Combustion Conditions on Toxicity

As described in Section 2.1, the Rx and Wild burns involved the same dry fuel bed composition but higher moisture content in Rx compared to Wild. This difference in moisture content induced differences in combustion conditions, most importantly, that duff was ignited in Wild but not in Rx, leading to overall lower combustion temperatures in Wild compared to Rx (Figure 2). Consequently, the smoke PM emissions in Rx had higher O/C, aromatics, and condensed aromatics compared to those in Wild (Figure 1 and Table 1). The difference in chemical composition of smoke PM between Rx and Wild led to differences in toxicity. Specifically, we found that the Rx smoke PM extracts increased cell death *via* necrosis, while the Wild smoke PM extracts did not significantly impact the release of LDH in the cell medium (Figure 4a). Although slightly more genes were dysregulated following the exposure to the Wild smoke PM extracts compared to the Rx smoke PM extracts (Figure 6), based on the cell death (Figure 4a), level of lipid peroxidation (Figure 5a), and magnitude of the gene fold-changes (Figure 6), our data suggest that the Rx smoke PM extracts were more toxic to BEAS-2B cells than Wild smoke PM extracts. We note, however, that other compositional differences that were not resolved in this study could contribute to the observed differences in toxicity. For example, Atwi et al.^32^ reported that in the addition to aromatic species, the effect of biomass-burning PM on metabolic activity was also correlated with levels of metals in the PM. It is possible that the Rx PM in this study contained higher levels of metals than the Wild PM. Nevertheless, our findings are consistent with previous studies that found that on a per-unit-mass basis, smoke emissions from high-temperature combustion induced more toxicity compared to low-temperature combustion.^17,74^

However, in general, the Rx and Wild smoke PM extracts induced similar patterns of effects (Figures 3–5). It is important to note that the mass loading of duff per unit area is typically 1 order of magnitude larger than surface fuels. Consequently, wildland fires that consume duff emit significantly higher levels of smoke per unit area burned compared with wildland fires that consume surface fuels only. Therefore, from a land-management and real-life exposure perspective, the public health burden of smoke emissions from a wildland fire that consumes duff will be significantly higher than that from a wildland fire that does not consume duff, as it will be driven by the difference in total amounts of smoke emissions rather than specific (per-unit-mass) toxicity.

### 4.3 Effect of Photochemical Aging on Toxicity

As described in Section 3.1, photochemical aging of the smoke in the OFR resulted in a 75% increase in OA mass due to the formation of SOA, which led to a substantial increase in oxygenated species (Figure 1) and a reduction in the relative abundance of aromatic and condensed aromatic species (Table 1). The difference in chemical composition of smoke PM between Wild and Wild-Aged led to differences in toxicity. Specifically, we found that Wild smoke PM extracts induced greater oxidative effects in BEAS-2B cells compared to Wild-Aged smoke PM extracts, as noted with the significantly lower concentration of 8-isoprostane in the cell media following Wild-Aged smoke PM extract exposure compared to Wild smoke PM extracts (Figure 5). The reduction in oxidative potential associated with photochemical aging observed in this study is consistent with previous studies that observed a reduction in the oxidative potential of smoke PM^30^ due to atmospheric photochemical aging.

PAHs, which are abundant in smoke PM, are ligands to the AHR, a transcription factor upstream of the xenobiotic response elements (XRE) signaling.^75^ PAHs are hydrophobic chemicals, and although the XRE pathway was activated by the smoke PM extracts in our study, with a higher responses seen following the Wild-Aged compared to the Wild smoke PM exposure (Figure 6), the hydrophobic PAHs content may have reached the surface of the cells and triggered the activation of the XRE pathway but may not have been internalized in the cells, which could potentially explain why we saw either no change or downregulation of the expression of genes associated with inflammation (Figure 6). In congruence with other studies, it was previously shown that CYP1A1 and AHRR were upregulated in airway epithelial cells following woodsmoke exposures.^76^ Thus, at the molecular level, Wild-Aged smoke PM extracts dysregulated the expression of biotransformation related genes, *e.g*., XRE signaling, to a greater extent than Wild smoke PM extracts (Figure 6). Furthermore, even though the differences were not statistically significant at the exposure dose in this study, the release of LDH in the cell media of BEAS-2B cells was higher for Wild-Aged than for Wild-Aged (Figure 5a), whereas the count of Caspase 3-positive cells was higher in Wild-Aged than in Wild (Figure 5b). These findings are consistent with our previous study, which showed that photochemical aging of smoke PM in a smog chamber led to a decrease of its effect on metabolic activity, but an increase in its effect on cell death by apoptosis.^32^ Our apoptosis results for Wild-Aged are also consistent with those of Liu et al.,^77^ who reported a significant dose-dependent increase in Caspase 3/7 activity induced by exposure to naphthalene-derived SOA. Overall, these results indicate that atmospheric aging alters the toxicity mechanisms induced by smoke PM.

### 4.4 Limitations and Future Directions

Our study has a few limitations, including the fact that we only used one dose (50 μg/mL) and one exposure/collection time point (24 h post exposure), thus limiting our ability to investigate time–course and dose–response relationships for the biological outcomes that we evaluated. However, dose–response relationships are necessary to infer causality, which has been previously established by numerous previous studies.^32,67,69,78^ Also, as in any cell culture experiment, cell viability data can be influenced by the presence of dead cells. Another limitation is the use of extracts. The extracts used for these exposures reflect mainly the particulate phase of the WFS emissions since we generated smoke PM extracts.^79^ As intended, these extracts did not capture the gas phase of the WFS emissions, which may also contribute significantly to the toxicity. Extracts are commonly used as *in vitro* methods to expose cells to combustion derived aerosols, including cigarette smoke and WFS; however, the extracts do not recapitulate the complexity of the aerosols in their entirety, including the compounds found in both the gas and particulate phases. Also, exposure of lung epithelial cells under submerged conditions is not physiologically representative of the lung deposition and clearance mechanisms, which could be simulated in a more relevant manner when using air–liquid interface (ALI) *in vitro* models. In addition, *in vitro* models do not recapitulate the complexity of whole-organism interactions and responses as would *in vivo* models. Current and future studies from our research group include *in vitro* ALI and *in vivo* (mice) exposure to direct wildland smoke emissions generated under various exposure conditions. Finally, the photochemical aging in this study was performed using an OFR, which does not fully represent photochemical aging in the atmosphere. The OFR simulates atmospheric OH exposure using high OH concentrations over short time scales, as opposed to low OH concentrations over long time scales in the atmosphere, and does not account for the full range of oxidation pathways that take place in the atmosphere.^80^

## 5 Conclusions

This study explored the effects of key variables associated with wildland-fire smoke emissions (prescribed fires versus drought-induced wildfires and fresh smoke versus photochemically aged smoke) on *in vitro* toxicity in human bronchial epithelial cells. Our results suggest a correlation between the chemical profiles of the organic portion of wildland-fire smoke particulate emissions, namely, the abundance of aromatic species, and the toxicity profiles toward BEAS-2B cells. We found that the higher temperatures associated with the combustion of surface fuels were associated with the production of smoke with enhanced toxicity compared to duff combustion. However, it is important to note that even though the smoke produced from the combustion of surface fuels is more toxic on a per-unit-mass basis, fires that consume duff typically produce significantly more smoke than fires that consume surface fuels only, which should be taken into consideration when assessing health impacts associated with smoke emissions from these two types of wildland fires. Furthermore, photochemical aging led to loss of oxidative potential and consequently diminished the smoke toxicity, using oxidative stress as a metric; however, there is a possibility that photochemical aging can enhance the smoke’s ability to induce cell death by apoptosis. These findings highlight the need to take into consideration combustion conditions and photochemical age of wildland-fire smoke when evaluating its toxicity.

## #

Aerodyne Research Inc., Billerica, Massachusetts 01821, USA

## ∇

Tofwerk USA, Boulder, Colorado 80301, USA.
